# The associations between water and sanitation and hookworm infection using cross-sectional data from Togo's national deworming program

**DOI:** 10.1371/journal.pntd.0006374

**Published:** 2018-03-28

**Authors:** Julia M. Baker, Victoria Trinies, Rachel N. Bronzan, Ameyo M. Dorkenoo, Joshua V. Garn, Sêvi Sognikin, Matthew C. Freeman

**Affiliations:** 1 Department of Epidemiology, Rollins School of Public Health, Emory University, Atlanta, Georgia, United States of America; 2 Department of Environmental Health, Rollins School of Public Health, Emory University, Atlanta Georgia, United States of America; 3 Health and Development International, Newburyport, Massachussetts, United States of America; 4 Ministère de la Santé et de la Protection Sociale, Lomé, Togo; Evidence Action, AUSTRALIA

## Abstract

**Background:**

Sustainable control of soil-transmitted helminths requires a combination of chemotherapy treatment and environmental interventions, including access to safe drinking water, sufficient water for hygiene, use of clean sanitation facilities, and handwashing (WASH). We quantified associations between home-, school-, and community-level WASH characteristics and hookworm infection—both prevalence and eggs per gram of stool (intensity)—among Togolese school children in the context of community-based chemotherapy treatments administered in the country from 2010 through 2014.

**Methodology/Principal findings:**

We analyzed data from two surveys conducted by the Togo Ministry of Health: a school-based survey of students aged 6–9 years across Togo conducted in 2009 and a follow-up survey in 2015, after four to five years of preventive chemotherapy. Data were available for 16,473 students attending 1,129 schools in 2009 and for 16,890 students from 1,126 schools in 2015. Between surveys, children in study schools received 0 to 8 rounds of deworming chemotherapy treatments. Few WASH conditions (only unimproved drinking water) were found to be significantly associated with the presence or absence of hookworms in an individual; however, quantitative eggs per gram of feces was associated with availability of unimproved drinking water, availability of improved drinking water either on or off school grounds, having a handwashing station with water available, and access to a sex-separate non-private or private latrine. The association between school WASH conditions and hookworm infection or burden often depended on the 2009 prevalence of infection, as more WASH characteristics were found to be significant predictors of infection among schools with high underlying endemicity of hookworm.

**Conclusions/Significance:**

Our findings emphasize the complex and often inconsistent or unpredictable relationship between WASH and hookworm. Specifically, we found that while preventive chemotherapy appeared to dramatically reduce hookworm infection, WASH was associated with infection intensity.

## Author summary

Preventive chemotherapy plays a critical role in breaking transmission of soil-transmitted helminths but it is likely that sustainable control of soil-transmitted helminths will require environmental improvements such as access to water for hygiene and hygienic sanitation, access to and use of a clean toilet facility, and handwashing with soap at key times (WASH). Here, we report on school-, home- and community-level WASH and the relationship of these characteristics with the prevalence and intensity of hookworm infection, one of the most prevalent soil-transmitted helminths, observed in the context of preventive chemotherapy among Togolese school children. In this large, country-wide assessment, few WASH conditions were found to be significant predictors of the presence or absence of hookworms in an individual, however, intensity of infection was associated with several WASH characteristics. Our analysis emphasizes the complex and often unpredictable role of WASH characteristics on hookworm, particularly relevant for Togolese and other international policy makers aiming to understand the interaction between WASH, preventive chemotherapy and hookworm. Further research taking into account not only WASH conditions but also use of WASH resources and practices would further strengthen our understanding of the interaction between WASH and deworming treatment as effective hookworm control strategies.

## Introduction

The burden of soil-transmitted helminths (STHs) remains a major public health concern in many parts of the world [1]. The most prevalent STH species in human infections include the roundworm (*Ascaris lumbricoides*), whipworm (*Trichuris trichiura*) and hookworm (*Necator americanus* and *Ancylostoma duodenale*) [1]. Control of hookworm presents a unique challenge; compared to many other STHs which infect humans through ingestion of eggs, infection with hookworm typically occurs through dermal contact with larvae in contaminated soil [2]. Though hookworm infection peaks in adults, school-age children bear a considerable burden of infection and morbidity [3]. The current World Health Organization (WHO) policy for the control of moderate to heavy infection is annual or biannual deworming for school-age children [4]. This preventive chemotherapy (PC) is typically done with benzimidazoles, which have been shown to be both safe and efficacious for hookworm reduction [5]. While treatment will immediately reduce the presence and intensity of infection, infection rates often return to pre-treatment levels within the span of several months [6].

Efforts are underway to assess the role of PC in breaking transmission, but it is likely that sustainable control of STHs will require environmental improvements such as access to sufficient water for hygiene and hygienic sanitation, access to and use of a clean toilet facility, and handwashing with soap at key times (WASH). The use of treated water, access to sanitation facilities, and soap use/availability have been shown to be associated with substantial reductions in the likelihood of any STH infection, represented by odds ratios (ORs) of 0.46 (95% confidence interval (CI): 0.36–0.60), 0.66 (95% CI: 0.57, 0.76) and 0.53 (95% CI: 0.29, 0.98), respectively [7]. One randomized trial in western Kenya assessed the impact of a school-based comprehensive WASH intervention on STH reinfection after school-based PC and found reduced reinfection rates in *Ascaris lumbricoides*, but not hookworm [8]. An assessment of the Kenya national deworming program revealed the association between handwashing and *Ascaris lumbricoides* infection depended on access to improved water at school and home for hygiene [9]. Similar evidence of the joint effects of school and community WASH access would support policy-relevant decisions on coordination of WASH in the context of neglected tropical disease (NTD) control programs [10–12].

In Togo, hookworm and other STHs continue to afflict the country’s population and present challenges for prevention and control [13]. Our analysis—focused on the association between WASH and STH infection—was nested within the Togo Ministry of Health’s survey to assess the impact of large scale community-based PC on the burden of STH in the country [12]. While data on other aspects of these efforts will be presented elsewhere, our research focused on hookworm, the STH with the highest prevalence of infection among Togolese school children at the conclusion of the study period. Our study assessed the role of school WASH access in enhancing the success of chemotherapy treatment on hookworm burden. Here, we report on school-, home- and community-level WASH characteristics and the relationship of these characteristics with reductions in the prevalence and intensity of hookworm infection observed after four to five years of PC in Togo.

## Methods

### Ethics statement

Ethical approval for this study was provided by the Togo Ministry of Health. The study was considered exempt from review by the Ethics Review Committee of Emory University as data were provided to the University de-identified. All students who agreed to participate were provided with a consent letter for their parents/guardians to review and accept. Parents/guardians provided informed consent on behalf of all child participants.

### Study design

This cross-sectional study assessed the association between WASH and hookworm infection using data collected from Togolese school children. STH mapping, led by the Togo Ministry of Health and partners, was conducted from October to December of 2009 as part of a nationwide NTD survey to evaluate the burden of STH, schistosomiasis and trachoma. Detailed baseline survey methods can be found elsewhere [14]. Briefly, data were collected from 35 districts across all five regions of the country, excluding the capital area of Lomé where very limited to no NTD transmission was suspected. Within these districts, all 632 sub-districts (each comprised of 10 or fewer villages) were surveyed in an effort to develop a nationally representative dataset. Two schools were selected from each sub-district, including one randomly selected school and one school suspected of high NTD prevalence. A total of 15 children between the ages of 6 and 9 years were selected by a teacher at each participating school via convenience sampling of the children who provided signed consent forms. Stool samples were collected from each student and analyzed at the field team’s mobile laboratory using Kato-Katz [15] to identify children with STH infection and determine the number of hookworm, roundworm (*Ascaris lumbricoides*), and whipworm (*Trichuris trichiura*) eggs per gram of stool (EPG). Our analysis focuses on hookworm infection due to its higher prevalence in the population.

During the five and a half years between the two assessments, house-to-house deworming treatments with albendazole were administered by the Togo Ministry of Health throughout the country, either annually or biannually as determined by WHO guidelines [16] and based on the district-level burden of disease in 2009. The treatments were administered at the district level such that every child in a given district (and thus every student) was targeted to receive the same number of treatments.

To assess the current STH burden and evaluate the effectiveness of PC, surveys were conducted from February to March 2015 in the same schools included in the 2009 evaluation with similar sampling methodologies. If a school surveyed in 2009 was no longer available for inclusion in 2015, the nearest school geographically was selected. At each school, a sample of 30 children aged 6 to 9 years were given consent forms the day before the study. Of those who returned with signed consent forms, 15 students were selected to participate in the study and provide a stool sample. Stool samples were examined using Kato-Katz [15] on site by laboratory technicians trained in the study protocol and intensity of infection was calculated as the number of hookworm EPG of stool. The schools’ global positioning system (GPS) coordinates were recorded using a hand-held GPS device and were linked with climate and population data (available at a spatial resolution of 1 km or finer) for that location using QGIS 2.14.5.

During the 2015 survey, data on school-level and individual-level WASH conditions, the primary predictors of interest, were collected via questionnaire. School conditions were reported during structured interviews with school staff and included questions related to the availability, source, and treatment of drinking water. Sanitation and hygiene conditions were observed directly, including an assessment of each latrine on the school grounds and availability of handwashing stations. Participating students were asked about WASH conditions in their homes using structured surveys. These individual-level data were also used to estimate community coverage of toilets and improved drinking water. Additional WASH conditions, such as open defecation on school grounds, were assessed by observation during the site visit.

### Statistical analysis

Our primary outcomes of interest were 1) individual-level presence or absence of eggs, a binary variable, and 2) individual-level EPG of feces, a count variable with a skewed distribution. Basic demographic and descriptive statistics are presented at the regional level for the 2009 and follow-up surveys. We also explored hookworm infection using several descriptive methods, including the percent of students testing positive for hookworm averaged across schools, average school-level EPG of stool, and individual-level EPG of stool using WHO categorizations of light, moderate and heavy [16]. Lastly, we described WASH conditions at the school- and individual-level.

We included a number of predictor variables in our models. Indicator variables were used to account for multi-category nominal WASH variables, including drinking water availability and source at school (none, unimproved, improved and available off school grounds, improved and available on school grounds), handwashing station access at school (none or station without water, station with water but no soap/ash, station with water and soap/ash), and latrine access at school (none available to pupils or not sex separate, at least one sex separate but not private latrine, at least one sex separate and private latrine). Climate and population data were included in the models to account for background factors that may influence the underlying endemicity of hookworm and WASH characteristics. These variables included the population density in 2015, distance to water bodies, distance to rivers, average annual minimum, maximum and mean temperature, average precipitation, land cover, and vegetation index linked to individual schools via GPS coordinates. All initial variables were selected based on previous literature, with population and climate variables narrowed to those significantly associated with prevalence of infection in bivariate analysis. Multicollinearity was assessed using Variance Inflation Factors (VIFs); variables with VIFs above 10 were removed from the models.

Outcomes were modeled using generalized estimating equations (GEE), accounting for clustering among students within a given school and including several pre-defined covariates. Presence of hookworm was evaluated using logistic regression to produce odds ratios (ORs) with significance testing using the Wald test, controlling for the number of community-based PC treatments received, 2009 hookworm prevalence, reported school-based deworming treatment in the last 12 months, district and relevant population/climate characteristics. We controlled for the prevalence of school-level hookworm infection in 2009, calculated among the 15 students in each school, as a proxy for individual baseline prevalence. Individual-level hookworm EPG was modeled using negative binomial regression, again controlling for the number of treatments, school-level 2009 hookworm prevalence, deworming treatments, district as well as population/climate characteristics. Negative binomial regression was used to estimate the EPG ratio, which can be described as the ratio of the expected counts of hookworm EPG for a one-unit change in the predictor variable or the relative change in mean intensity of hookworm infection for a one-unit change in the predictor variable.[17] Exchangeable correlation structures were assumed for each model type in an effort to account for correlation among individuals at the same school.

The models described above were each applied to the entire population and three population subsets—1) schools with a low underlying endemicity of hookworm (less than 20%), 2) schools with a high underlying endemicity of hookworm (20% or more), and 3) schools with students who did not receive any school or community-based PC treatment in the previous 12 month period. In a separate analysis, we ran the logistic and negative binomial models for all data stratified by the number of house-to-house chemotherapy treatments received. These models used the same outcome and predictor variables previously described with the exception of the number of chemotherapy treatments, which was excluded as a predictor variable in the model. Separate models were run for both outcomes and at each level of treatment. For all models, individuals missing data were excluded. General model statements for prevalence and intensity of hookworm infection are shown in S1 Model Statements.

We assessed the role of community sanitation coverage on hookworm prevalence to assess potential indirect herd protection as has been modeled and found empirically elsewhere [9, 18, 19]. Two community-level WASH variables were created by aggregating individual-level home latrine access and water source data to estimate the proportion of the community with access to a latrine at home and the proportion with access to improved drinking water, respectively. The relationship between these community-level WASH coverage variables and intensity of hookworm infection were modeled using an unadjusted simple linear cubic spline.

Data were collected using Open Data Kit and analyzed using STATA v.14 (College Station, TX).

## Results

Data from the Togo Ministry of Health surveys were available for 33,363 students, including 16,473 students from 1,129 schools in 2009 and 16,890 students from 1,126 schools in 2015 (Table 1). The number of schools surveyed in 2009 and at follow-up remained nearly constant for each of the five regions. Data on hookworm infection were available for nearly all children surveyed, including 16,090 (97.7%) students at baseline and 16,887 (>99.9%) at follow-up. The mean age of children at baseline was 7.7 years (range: 6–9) and 40.6% were females. At follow-up, the mean age of children was 8.2 (range: 6–9) and the sex distribution remained slightly skewed towards males (46.6% female).

**Table 1:** Study population by region.

	Savanes	Kara	Centrale	Plateaux	Maritime	Total
**Schools surveyed, total**						
2009	134	235	164	375	221	1129
2015	134	233	164	375	220	1126
**Children surveyed, total number (mean per school)**						
2009	2010 (15.0)	3063 (14.9)	2460 (15.0)	5625 (15.0)	3315 (15.0)	16473 (15.0)
2015	2010 (15.0)	3495 (15.0)	2460 (15.0)	5625 (15.0)	3300 (15.0)	16890 (15.0)
**Percent female, mean**						
2009	38.1	42.1	41.6	40.1	40.7	40.6
2015	44.9	45.5	45.5	46.9	49.6	46.6
**Student age, mean (sd)**						
2009	7.7 (0.9)	7.5 (1.1)	7.7 (2.1)	7.6 (1.0)	7.8 (1.1)	7.7 (1.3)
2015	8.3 (0.8)	8.2 (0.9)	8.2 (0.8)	8.4 (0.8)	7.9 (1.1)	8.2 (0.9)

### Hookworm burden

Hookworm prevalence and intensity of infection were categorized according to WHO guidelines [20], along with the number of treatments received (Table 2). Over the six-year period, children received an average of 3.8 PC treatments. The average school-level prevalence of hookworm decreased from 32.4% in 2009 to 11.1% in 2015. Concurrently, the average school-level EPG of stool among students decreased from a mean of 184.7 eggs/g to 33.1 eggs/g. In 2015, fewer than 11% of children had light infection. Moderate and heavy infection was 1.8% in 2009 and 0.3% in 2015, which meets the WHO target of <1%. Throughout the study period, the intensity of infection differed by sex. In 2009, females had an average of 130.7 eggs/g (95% CI: 114.8–146.5) while males had a substantially higher average of 221.9 eggs/g (95% CI: 201.2–242.7). This relationship stayed consistent in 2015, as females had a lower average number of eggs/g (22.1 eggs/g, 95% CI: 18.3–26.0) than males (42.8 eggs/g, 95% CI: 36.7–48.9).

**Table 2:** Prevalence and intensity of hookworm infections in 2009 and 2015 and number of treatments.

	Savanes (2009 n = 1930; 2015 n = 2009)	Kara (2009 n = 2968; 2015 n = 3494)	Centrale (2009 n = 2446; 2015 n = 2460)	Plateaux (2009 n = 5491; 2015 n = 5624)	Maritime (2009 n = 3255; 2015 n = 3300)	Total (2009 n = 16090; 2015 n = 16887)
**Average school percent positive for hookworm [range]**^**†**^						
2009	31.8 [0.0, 100.0]	24.2 [0.0, 100.0]	36.4 [0.0, 100.0]	31.7 [0.0, 100.0]	38.5 [0.0, 100.0]	32.4 [0.0, 100.0]
2015	4.6 [0.0, 40.0]	9.5 [0.0, 66.7]	15.2 [0.0, 66.7]	13.0 [0.0, 100.0]	10.3 [0.0, 53.3]	11.1 [0.0, 1.0]
Average school-level hookworm EPG* **of stool, arithmetic mean [range]**						
2009	331.5 [0–36864]	72.6 [0, 12240]	205.0 [0, 36000]	169.4 [0, 17808]	210.6 [0, 20544]	184.7 [0, 36864]
2015	7.9 [0, 5664]	22.6 [0, 7056]	37.2 [0, 6360]	43.3 [0, 11952]	39.2 [0, 4944]	33.1 [0, 11952]
Individual-level hookworm EPG* **of stool, frequency**^**§**^ **(%)**						
2009						
None	1316 (68.2)	2251 (75.8)	1555 (63.6)	3753 (68.3)	2003 (61.5)	10878 (67.6)
Light	541 (28.0)	703 (23.7)	844 (34.5)	1637 (29.8)	1190 (36.6)	4915 (30.5)
Moderate	35 (1.8)	10 (0.3)	28 (1.1)	56 (1.0)	35 (1.1)	164 (1.0)
Heavy	38 (2.0)	4 (0.1)	19 (0.8)	45 (0.8)	27 (0.8)	133 (0.8)
2015						
None	1916 (95.4)	3161 (90.5)	2086 (84.8)	4895 (87.0)	2961 (89.7)	15019 (88.9)
Light	92 (4.6)	326 (9.3)	367 (14.9)	708 (12.6)	323 (9.8)	1816 (10.8)
Moderate	0 (0.0)	6 (0.2)	5 (0.2)	15 (0.3)	12 (0.4)	38 (0.2)
Heavy	1 (0.0)	1 (0.0)	2 (0.1)	6 (0.1)	4 (0.1)	14 (0.1)
**Number of treatments, mean (range)**						
2010–2014	3.7 [0, 8]	2.2 [0, 5]	5.0 [5, 5]	4.0 [0, 7]	4.4 [4, 7]	3.8 [0, 8]

^**§**^Hookworm categorization based on WHO guidelines. None = 0 eggs/g, Light = 1–1,999 eggs/g, Moderate = 2,000–3,999 eggs/g, Heavy = 4,000+ eggs/g.

^**†**^Mean percentage of school-level population (mean of means)

*EPG = eggs per gram of stool

### WASH conditions

A mix of WASH conditions were observed among the five regions, with no one region demonstrating consistently better WASH conditions relative to others (Table 3). Less than half (45.3%) of schools had improved drinking water available, defined as water from a tap/standpipe, bore well, covered well, or rain water. Few schools (3.5%) had treated their water in the last two weeks. Open defecation remained prevalent throughout the country, particularly in the Kara region, where it was observed in nearly three-quarters of schools. At a majority of schools (60.7%), pupils had access to at least one latrine, though access to a sex separate, private latrine was much less common (25.2%). Availability of a hand washing station varied by region, ranging from 2.2% in Savanes to 22.7% in Plateaux.

**Table 3:** School and home WASH conditions.

WASH characteristic	Savanes	Kara	Centrale	Plateaux	Maritime	Total
**School WASH conditions**						
Improved drinking water available*, number (%)	47 (35.1)	109 (46.8)	99 (60.4)	157 (41.9)	99 (45.0)	511 (45.4)
Water located on school grounds, number (%)	35 (26.1)	58 (24.9)	87 (53.1)	72 (19.2)	67 (30.5)	319 (28.3)
Water treated in past 2 weeks, number (%)	1 (0.8)	6 (2.6)	4 (2.4)	25 (6.7)	3 (1.4)	39 (3.5)
Open defecation during the school day, number (%)	49 (36.6)	171 (73.4)	100 (61.0)	149 (39.7)	98 (44.6)	567 (50.4)
At least 1 latrine available for pupils, number (%)	110 (82.1)	169 (72.5)	122 (74.4)	177 (47.2)	107 (48.6)	685 (60.8)
Sex separate, private latrines available, number (%)	43 (32.1)	50 (21.5)	49 (29.9)	77 (20.5)	65 (29.6)	284 (25.2)
Hand washing station available (w/ water & soap/ash), number (%)	3 (2.2)	10 (4.3)	13 (7.9)	85 (22.7)	10 (4.6)	121 (10.8)
**Home/child WASH conditions**						
Improved drinking water available, number (%)	892 (44.4)	2464 (70.5)	1774 (72.1)	2573 (45.7)	1646 (49.9)	9349 (55.4)
Latrine/WC available, number (%)	504 (25.1)	613 (17.5)	888 (36.1)	2021 (35.9)	1566 (47.5)	5592 (33.1)
Child wearing shoes, number (%)	1424 (70.9)	2594 (74.2)	1790 (72.8)	4010 (71.3)	2420 (73.3)	12,238 (72.7)

*Improved drinking water categorized as water from a tap/standpipe, borewell, covered well, or rain water

Home WASH conditions were more consistent across regions, with more than half of students having access to improved drinking water at home and about one-third having access to a latrine or WC. Nearly three-quarters of the students were observed to be wearing shoes.

Data collection could not be completed on school grounds for 47 schools because of a teachers’ strike that occurred during data collection. For these schools, data on school WASH conditions were unavailable and information on students’ home WASH conditions were gathered via student interviews at the time of stool sample collection.

**Fig 1:**
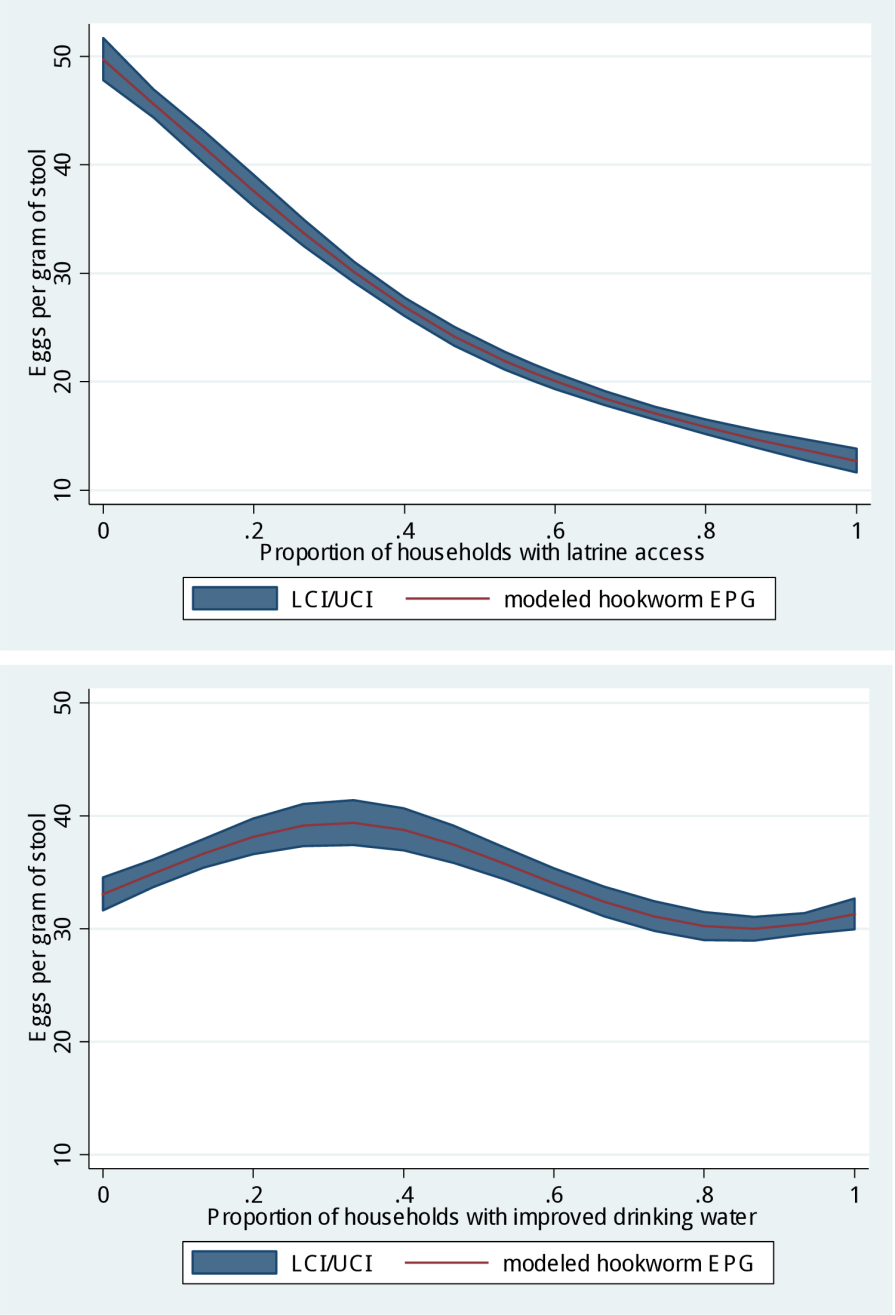
Relationship between individual-level intensity of infection (eggs per gram of stool) and community-level WASH characteristics.

The spline model for community-level latrine coverage showed lower EPG of stool with increasing latrine coverage that was approximately linear (Fig 1). We did not observe a clear, linear trend between improved community-level drinking water and EPG of stool.

### Multivariable associations

Several WASH conditions were associated with odds of hookworm infection (presence/absence of eggs) and particularly with intensity of infection (EPG of stool, Table 4). Compared to no drinking water at school, availability of unimproved drinking water was associated with higher odds of infection and intensity of infection (OR = 1.40, 95% CI: 1.09–1.80; EPG ratio = 1.20, 95% CI: 1.13–1.28). Access to improved drinking water, either off or on school grounds, was not associated with the prevalence of hookworm compared to no drinking water (OR = 0.90, 95% CI: 0.71–1.14; OR 1.14, 95% CI: 0.92–1.42). We found that access to improved drinking water off school grounds was associated with lower intensity of infection compared to no access (EPR ratio = 0.80, 95% CI: 0.76–0.85), whereas having access to improved drinking water on school grounds was associated with higher intensity of infection (EPG ratio = 1.50, 95% CI: 1.42–1.58). Neither handwashing stations (with water OR = 0.85, 95% CI: 0.57–1.29; with water and soap/ash OR = 0.77, 95% CI: 0.57–1.05) nor latrine access at school (sex separate, non-private latrine OR = 0.84, 95% CI: 0.52–1.36; sex separate, private latrine OR = 0.93, 95% CI: 0.77–1.14) was associated with prevalence of infection. In contrast, having a handwashing station with water (and no soap/ash) and having access to a sex separate, private latrine were associated with lower intensity of infection (EPG ratio = 0.79, 95% CI: 0.71–0.87; EPG ratio = 0.90, 95% CI: 0.86–0.95). Shoe wearing was associated with lower prevalence and intensity of infection (OR = 0.69, 95% CI: 0.62–0.78; EPG ratio = 0.46, 95% CI: 0.44–0.48).

**Table 4:** Multivariable associations between WASH conditions and prevalence/intensity of hookworm infection.

	Prevalence of infection (presence/absence of eggs)			Intensity of Infection (eggs per gram of stool)		
Condition of interest	OR	95% CI	p-value	EPG ratio	95% CI	p-value
**WASH conditions**						
Water source and availability at school (categorical)						
No drinking water available*	ref	—	—	—	—	—
Unimproved drinking water available	1.40	1.09, 1.80	**0.01**	1.20	1.13, 1.28	**<0.01**
Improved drinking water available, not on school grounds	0.90	0.71, 1.14	0.37	0.80	0.76, 0.85	**<0.01**
Improved drinking water available, on school grounds	1.14	0.92, 1.42	0.22	1.50	1.42, 1.58	**<0.01**
Handwashing station availability at school (categorical)						
No handwashing station or station without water*	ref	—	—	—	—	—
Handwashing station available with water	0.85	0.57, 1.29	0.45	0.79	0.71, 0.87	**<0.01**
Handwashing station available with water and soap/ash	0.77	0.57, 1.05	0.10	1.05	0.98, 1.12	0.17
Latrine availability and type at school (categorical)						
No latrine or not sex separate*	ref	—	—	—	—	—
Sex separate, non-private latrine available	0.84	0.52, 1.36	0.48	0.92	0.82, 1.04	0.19
Sex separate, private latrine available	0.93	0.77, 1.14	0.49	0.90	0.86, 0.95	**<0.01**
Child wearing shoes	0.69	0.62, 0.78	**<0.01**	0.46	0.44, 0.48	**<0.01**
**School-level background variables**						
2009 hookworm prevalence	6.04	4.30, 8.48	**<0.01**	9.23	8.47, 10.06	**<0.01**
Number of deworming treatments	0.93	0.88, 0.97	**<0.01**	0.90	0.89, 0.91	**<0.01**
Deworming treatment in the last 12 months	1.23	1.04, 1.45	**0.02**	1.18	1.13, 1.23	**<0.01**

* Reference category. Models control for 2015 population density, distance from school to water, district, and land cover.

Bold p-values are statistically significant at the α = 0.05 level

Among the community-level WASH variables explored, neither improved drinking water nor latrine coverage was included in the models due to multicollinearity.

The significance of the 2009 prevalence of hookworm motivated several sensitivity analyses performed on subsets of the population. When limited to students at schools with a low prevalence of hookworm infection at baseline (n = 5,594, prevalence less than 20%), none of the school or individual WASH conditions were found to be statistically significantly associated with the odds of infection except for school-level access to an unimproved water source (OR: 1.73, 95% CI: 1.07, 2.80). The equivalent model for intensity of infection could not be assessed because of non-convergence.

Among students at schools with a high prevalence of infection at baseline (n = 10,124, prevalence of 20% or more), access to improved drinking water on school grounds, compared to no drinking water, was associated with a higher odds of infection (OR = 1.35, 95% CI: 1.01–1.80), while shoe wearing was associated with a lower odds of infection (OR = 0.65, 95% CI: 0.57–0.74). No other predictors were statistically significantly associated with the outcome. For intensity of infection, patterns of significance were similar to those observed when modeling all schools.

Multivariable associations were assessed among children at schools that neither received community-based PC nor reported any school-based PC in the previous 12 months (n = 2,009). None of the primary WASH predictors were associated with prevalence of infection. We were unable to determine associations for intensity of infection due to non-convergence of the model.

Lastly, the prevalence and intensity of hookworm infection were modeled separately for children at schools in districts that received 0–1, 4–5, and 7–8 house-to-house treatments (no children received 2–3 or 6 treatments). We were unable to assess intensity of infection among children living in districts that received 0–1 house-to-house treatments because of non-convergence of the model. Several similar results to those described above for all children combined were observed for the stratified analyses for both prevalence (S1 Table) and intensity of infection (S2 Table) with few statistically significant associations in the former and more in the latter. Availability of improved drinking water on school grounds was associated with higher intensity of infection when compared to no drinking water at school (4–5 treatments: EPG ratio = 1.46, 95% CI: 1.37–1.55; 7–8 treatments: EPG ratio = 1.47, 95% CI: 1.30–1.66). As with all schools combined, neither handwashing nor latrine access was significantly associated with prevalence of infection for any level of treatment. Notably, shoe wearing was associated with a lower intensity of infection with the EPG ratio decreasing as the number of treatments increased (EPG ratio = 0.44, 95% CI: 0.42–0.46 among children living in districts that received 4–5 home-based treatments; EPG ratio = 0.28, 95% CI: 0.25–0.31 among children living in districts that received 7–8 home-based treatments). When measuring the prevalence of infection, lower odds ratios were observed among schools in districts that received 4–5 and 7–8 house-to-house treatments (OR = 0.64 and 0.71, respectively) when compared to the odds ratio for schools in districts that received 0–1 house-to-house treatments, but only the odds ratio for schools in districts that received 4–5 house-to-house treatments showed statistical significance (OR = 0.64, 95% CI: 0.56–0.73).

## Discussion

The country-wide assessment of hookworm infection among Togolese school children before and after a national door-to-door deworming campaign provided a valuable opportunity for insight into the complex relationship between WASH conditions and hookworm in communities receiving PC. Results from the 2015 assessment indicate WASH conditions were poor for a majority of schools. Few WASH conditions were found to be significant predictors of the presence or absence of hookworms in an individual; however, intensity of infection was associated with several individual-, school- and community-level WASH characteristics, although the direction of the association was inconsistent.

Individual-, school-, and community-level WASH characteristics assessed in 2015 varied substantially across the five regions. Fewer than half of schools had improved drinking water available for pupils, nearly no schools had drinking water that had been treated in the last two weeks, a quarter of schools had at least one sex separate, private latrine available to pupils, and about one in 10 schools had a hand washing station with water and soap/ash. The assessment of home-based WASH conditions found that approximately half of the students had an improved drinking water source available, one-third had access to a latrine/WC and three-quarters were wearing shoes at the time of the survey. Shoe wearing at the time of survey was found to be associated with reduced intensity of infection in all population groups assessed, consistent with similar studies [7, 10]. Shoe wearing may modify the effect of deworming with larger impacts among individuals who received more treatments, as suggested by our analysis stratified by the number of treatments.

Existing literature demonstrates the need for nuanced analysis of water-related practices, including handling and storage, and its relationship with hookworm infection. Literature on the relationship between hookworm and water quality and source location is limited [7], often focusing on a specific water source and other STHs [7, 21, 22]. Based on the biology and life cycle of the parasite, the relationship between water and hookworm is likely due to the presence of sufficient water for personal hygiene and toilet cleaning, rather than “improved water” for drinking. Further, use of “inappropriate” water receptacles for water storage has been shown to increase the odds of STH infection in Venezuela [22]. Some evidence suggests that water acquired on site or from a private well increases the odds of infection [21, 23]. In our study, access to hand washing stations with water, with or without soap/ash, was not significantly associated with hookworm prevalence across all models; however, it was a significant, though inconsistent (in terms of the direction of the association), predictor of intensity of infection across models. It is possible that our findings are limited by our measure of presence of hand washing stations rather than actual observation of hand washing practice, as existing literature suggests hand hygiene is associated with lower odds of STH infection; however, this relationship is likely dependent on the timing and frequency of such behaviors [7].

As with handwashing stations, access to sex separate, private latrines was not associated with prevalence of infection but was often a predictor of intensity of infection, a more meaningful measure of morbidity [24]. In most models with EPG of stool as the outcome, at least one latrine predictor (access to sex separate, non-private latrine or access to sex separate, private latrine) was found to be significantly associated with lower intensity of infection. Both positive and negative associations between sanitation access and hookworm infection have been demonstrated in the literature [7] and many studies have found a non-significant relationship [25–28]. In general, associations found between school WASH characteristics and hookworm infections suggest that access to toilets and hand washing facilities may be important but not sufficient means for reducing infection and might also reflect critical limitations of our measures, primarily that they only address access and not use of the resources.

We found that the proportion of households with access to latrines in a school catchment had an approximately linear inverse relationship with intensity of hookworm infection, possibly indicating a measure of herd protection. Community coverage of improved drinking water, on the other hand, demonstrated a very different relationship with hookworm infection. The results of our bivariate analysis suggest that improved community coverage may reduce infection intensity among individuals. While hookworm is not typically thought to be spread through drinking water, improved access to water may improve the relevant hygiene behaviors [7]. This relationship holds until coverage reaches about 80% of the population at which time little change in intensity of infection occurs. A recent analysis of community sanitation coverage and trachoma found that coverage of 70% was the point in which sanitation had an impact; similar results were found at 80% for diarrhea [29].

A particularly salient finding from this analysis emerged when modeling hookworm prevalence among children at schools that had not received school- or community-based helminth treatment in the last 12 months. None of the WASH characteristics included in the model for this subgroup were significant, potentially indicative of the complex, sometimes inconsistent interaction between WASH resources and STH infection [7] and the short-term effects of PC demonstrated in the literature [30]. Alternatively, it could be that the reason they were not treated recently was because of perceived low prevalence and intensity of hookworm infection in these communities; over 80% of these school had a hookworm prevalence below 20% in 2009. These results may reflect the impacts of prophylaxis outside the national deworming program and reported school-based deworming or other limitations in data availability and statistical models.

Our finding emphasizes the importance of baseline data when planning and evaluating deworming treatment activities as this measure seems, by far, to be the largest predictor of the STH infection. The 2009 prevalence of hookworm was associated with the number of treatments by programmatic design and our data support an association between baseline prevalence and prevalence in 2015. Without controlling for baseline prevalence, results would likely be confounded by the level of endemicity. The number of treatments received by schools ranged from zero to eight, differing greatly by district, the implementation unit for PC. Our analysis found that the number of treatments was associated with lower 2015 hookworm prevalence and intensity of infection when analyzed among all schools combined.

Limitations of the included variables prevented more detailed analysis of the effect of treatment. The variables included in the analysis do not take into account the timing of each treatment and the outcome variables do not capture change in hookworm prevalence or intensity of infection. Such outcome assessment was not possible at the individual pupil level because the students from the original baseline evaluation were not the same as those surveyed at follow-up, prohibiting linkage of pre- and post-treatment data.

Other constraints of the study should be noted. This exploratory and hypothesis-generating study inherently included multiple testing, which raises the potential for spurious chance findings. As such, conclusions based on p-values should be carefully interpreted. The observational nature of the study and inability to measure hookworm in the same individuals at the surveyed schools over time restricts causal inference. Non-random selection of students and the narrow age range of the students is of concern, potentially limiting how well the underlying endemicity of hookworm at each school was captured, the representativeness of follow-up study participants and the study’s generalizability within Togo. Data collection constraints also prevented evaluation of several important confounders, such as the number of students per school, un-programmed deworming activities, WASH practices or WASH conditions at baseline and other measures of contact with soil. Estimates of community-level characteristics (water source and latrine use) are limited in that they were extrapolated from a small number of students’ reported household characteristics and should be interpreted cautiously. Lastly, this analysis does not take into effect the potential impact of mass treatments for lymphatic filariasis received in several communities prior to the 2009 survey. Six of the eight districts previously endemic for lymphatic filariasis received no PC from 2010 through 2014 because they had a hookworm prevalence below 20% in 2009. It is possible that the prior lymphatic filariasis treatment may have resulted in artificially low baseline prevalence estimates for hookworm, which could dilute our measure of the association between hookworm and WASH conditions.

Our analysis contributes to the growing body of literature on the potential importance of school- and community-level WASH characteristics on child health [18, 19, 31, 32]. Analyses of WASH access at the school and community levels using longitudinal data have been explored in Kenya, but only for *A*. *lumbricoides* [8, 9]. Our study contributes to existing literature and addresses several shortcomings often encountered when examining the association between STH infection and WASH conditions. As opposed to most research which has focused on WASH conditions in a single environment, the focus of our study was on both the school and household environments where school-age children split their time [7]. The inclusion of 2009 hookworm data and community-level WASH data further strengthens the study. A recent systematic review demonstrated that a majority of studies in this field are cross-sectional in design [7]. This study’s cross-sectional design is enhanced by incorporating previous hookworm measures at the school level to control for underlying endemicity and allows for control of this key confounder. Additional advantages result from the study’s large sample size, detailed information on WASH conditions, and data collection methods, which included both self-report and direct observation of WASH conditions. With these strengths, the study provides important policy-relevant information both specific to the Togo context and applicable beyond the country.

In summary, this school-based cross-sectional study provides insight into the impact of WASH on hookworm in the context of PC. These findings emphasize the complex, often unpredictable relationship between WASH and hookworm. The role of school WASH conditions on hookworm prevalence and intensity varied and often depended on the underlying endemicity of hookworm infection, with more WASH characteristics being associated with infection and disease burden among children in schools with a high underlying endemicity. Further research taking into account not only WASH conditions but also use of WASH resources and practices would further strengthen our understanding of the interaction between WASH and PC as effective hookworm control strategies.

## Supporting information

S1 STROBE Checklist (DOCX)

S1 Model Statements General model statements for prevalence and intensity of hookworm infection. (DOCX)

S1 Table Multivariable associations between WASH conditions and prevalence of hookworm infection (presence/absence of eggs in stool), stratified by number of house-to-house deworming treatments. (DOCX)

S2 Table Multivariable associations between WASH conditions and intensity of hookworm infection (eggs per gram of stool), stratified by number of house-to-house deworming treatments. (DOCX)
