# Supplementary material for: The associations between water and sanitation and hookworm infection using cross-sectional data from Togo's national deworming program
Source: PLoS Negl Trop Dis. 2018 Mar 28;12(3):e0006374. doi: 10.1371/journal.pntd.0006374 (PMC5902041; doi:10.1371/journal.pntd.0006374)
Supplement: S1 STROBE Checklist — (DOCX) [file pntd.0006374.s001.docx]

**S1. STROBE checklist**

STROBE Statement—Checklist of items that should be included in reports of *cross-sectional studies*

|  | Item No | Recommendation |
| --- | --- | --- |
| **Title and abstract** | 1 | (*a*) Pg 1: Cross-sectional mentioned in title |
|  |  | (*b*) Pg 2: Methodology/Principal Findings section summarizes the structure of the study and primary results |
| Introduction | | |
| Background/rationale | 2 | Pg 3-5  “The burden of soil-transmitted helminths (STHs) remains a major public health concern in many parts of the world.”  “Efforts are underway to assess the role of MDA in breaking transmission, but it is likely that sustainable control of STHs will require environmental improvements such as access to sufficient water for hygiene and hygienic sanitation, access to and use of a clean toilet facility, and handwashing with soap at key times (WASH).”  “In Togo, hookworm and other STHs continue to afflict the country’s population and present challenges for prevention and control.” |
| Objectives | 3 | Pg. 5  “Our study aims to assess the role of school WASH access in enhancing the success of mass chemotherapy treatment on hookworm burden.” |
| Methods | | |
| Study design | 4 | Pg. 5-7  This cross-sectional study used data from a 2009 and 2015 survey of Togolese school children to assess the association between WASH and hookworm infection.  “STH mapping, led by the Togo Ministry of Health and partners, was conducted from October to December of 2009 as part of a nationwide NTD survey to evaluate the burden of STH, schistosomiasis and trachoma.”  “Briefly, data were collected from 35 districts across all five regions of the country... Within these districts, all 632 sub-districts (each comprised of 10 or fewer villages) were surveyed in an effort to develop a nationally representative dataset. Two schools were selected from each sub-district, including one randomly selected school and one school suspected of high NTD prevalence. A total of 15 children between the ages of 6 and 9 years were selected by a teacher at each participating school. Stool samples were collected from each student and analyzed at the field team’s mobile laboratory using Kato-Katz to identify children with STH infection and determine the number of hookworm… eggs per gram of stool (EPG).”  “To assess the current STH burden and evaluate the effectiveness of mass treatments, surveys were conducted from February to March 2015 in the same schools included in the 2009 baseline evaluation.”  “During the 2015 survey, data on school-level and individual-level WASH conditions, the primary predictors of interest, were collected via questionnaire.” |
| Setting | 5 | Pg. 5-7  “This cross-sectional study assessed the association between WASH and hookworm infection using data collected from Togolese school children.”  “STH mapping, led by the Togo Ministry of Health and partners, was conducted from October to December of 2009 as part of a nationwide NTD survey to evaluate the burden of STH, schistosomiasis and trachoma.”  “Briefly, data were collected from 35 districts across all five regions of the country... Within these districts, all 632 sub-districts (each comprised of 10 or fewer villages) were surveyed in an effort to develop a nationally representative dataset. Two schools were selected from each sub-district... A total of 15 children between the ages of 6 and 9 years were selected by a teacher at each participating school.”  “To assess the current STH burden and evaluate the effectiveness of mass treatments, surveys were conducted from February to March 2015 in the same schools included in the 2009 baseline evaluation. If the school surveyed in 2009 was no longer available for inclusion, the nearest school geographically was selected. At each school, a sample of 30 children aged 6 to 9 years were given consent forms the day before the study.” |
| Participants | 6 | Pg. 5-6  In 2009: “A total of 15 children between the ages of 6 and 9 years were selected by a teacher at each participating school via convenience sampling of the children who provided signed consent forms.”  In 2015: “At each school, a sample of 30 children aged 6 to 9 years were given consent forms the day before the study. Of those who returned with signed consent forms, 15 students were selected to participate in the study and provide a stool sample.” |
| Variables | 7 | Pg 7-9  “Our primary outcomes of interest were 1) individual-level presence or absence of eggs, a binary variable, and 2) individual-level EPG of feces, a continuous, skewed variable.”  “Indicator variables were used to account for multi-category nominal WASH variables, including drinking water availability and source at school (none, unimproved, improved and available off school grounds, improved and available on school grounds), handwashing station access at school (none or station without water, station with water but no soap/ash, station with water and soap/ash), and latrine access at school (none available to pupils or not sex separate, at least one sex separate but not private latrine, at least one sex separate and private latrine). Climate and population data were included in the models to account for background factors that may influence the underlying endemicity of hookworm and WASH characteristics. These variables included the population density in 2015, distance to water bodies, distance to rivers, average annual minimum, maximum and mean temperature, average precipitation, land cover, and vegetation index linked to individual schools via GIS coordinates.”  “Two community-level WASH variables were created by aggregating individual-level home latrine access and water source data to estimate the proportion of the community with access to a latrine at home and proportion with access to improved drinking water, respectively.” |
| Data sources/ measurement | 8* | Pg. 6-9  “Stool samples were collected from each student and analyzed at the field team’s mobile laboratory using Kato-Katz to identify children with STH infection and determine the number of hookworm… eggs per gram of stool (EPG).”  “During the 2015 survey, data on school-level and individual-level WASH conditions, the primary predictors of interest, were collected via questionnaire. School conditions were reported during structured interviews with school staff, and included questions related to the availability, source, and treatment of drinking water. Sanitation and hygiene conditions were observed directly, including an assessment of each latrine on the school grounds and availability of handwashing stations. Participating students were asked about WASH conditions in their homes using structured surveys... . Additional WASH conditions, such as open defecation on school grounds, were assessed by observation during the site visit. ” |
| Bias | 9 | Pg. 5-6  “Within these districts, all 632 sub-districts (each comprised of 10 or fewer villages) were surveyed in an effort to develop a nationally representative dataset. Two schools were selected from each sub-district, including one randomly selected school and one school suspected of high NTD prevalence.”  “…surveys were conducted from February to March 2015 in the same schools included in the 2009 evaluation with similar sampling methodologies. If the school surveyed in 2009 was no longer available for inclusion, the nearest school geographically was selected.” |
| Study size | 10 | Pg. 5  “Briefly, data were collected from 35 districts across all five regions of the country, excluding the capital area of Lomé where very limited to no NTD transmission was suspected. Within these districts, all 632 sub-districts (each comprised of 10 or fewer villages) were surveyed in an effort to develop a nationally representative dataset. Two schools were selected from each sub-district, including one randomly selected school and one school suspected of high NTD prevalence. A total of 15 children between the ages of 6 and 9 years were selected by a teacher at each participating school.” |
| Quantitative variables | 11 | Pg. 7-9  “Indicator variables were used to account for multi-category nominal WASH variables, including drinking water availability and source at school (none, unimproved, improved and available off school grounds, improved and available on school grounds), handwashing station access at school (none or station without water, station with water but no soap/ash, station with water and soap/ash), and latrine access at school (none available to pupils or not sex separate, at least one sex separate but not private latrine, at least one sex separate and private latrine).”  “All initial variables were selected based on previous literature, with population and climate variables narrowed to those significantly associated with prevalence of infection in bivariate analysis. Multicollinearity was assessed using Variance Inflation Factors (VIFs); variables with VIFs above 10 were removed from the models.” |
| Statistical methods | 12 | Pg. 7-9  (*a*) Describe all statistical methods, including those used to control for confounding |
|  |  | (*b*) “Climate and population data were included in the models to account for background factors that may influence the underlying endemicity of hookworm and WASH characteristics.”  “Multicollinearity was assessed using Variance Inflation Factors (VIFs)”  “Outcomes were modeled using generalized estimating equations (GEE), accounting for clustering among students within a given school and including several pre-defined covariates. Presence of hookworm was evaluated using binomial logistic regression with significance testing using the Wald test…”  “Individual-level hookworm EPG was modeled using negative binomial logistic regression, again controlling for the number of treatments, school-level 2009 hookworm prevalence, deworming treatments, district as well as population/climate characteristics. Exchangeable correlation structures were assumed for each model type in an effort to account for correlation among individuals at the same school.”  “The models described above were each applied to the entire population and three population subsets—1) schools with a low underlying endemicity of hookworm (less than 20%), 2) schools with a high underlying endemicity of hookworm (20% or more), and 3) schools with students who did not receive any school or community-based hookworm mass treatment in the previous 12 month period.” |
|  |  | (*c*) “Individuals missing data were excluded from the models.” |
|  |  | (*d*) not applicable |
|  |  | (*e*) Describe any sensitivity analyses  Pg. 9, 15  “The significance of the 2009 prevalence of hookworm motivated several sensitivity analyses performed on subsets of the population.”  “The models described above were each applied to the entire population and three population subsets—1) schools with a low underlying endemicity of hookworm (less than 20%), 2) schools with a high underlying endemicity of hookworm (20% or more), and 3) schools with students who did not receive any school or community-based hookworm mass treatment in the previous 12 month period.”  “In a separate analysis, we ran the logistic and negative binomial models for all data stratified by the number of house-to-house mass deworming treatments received. These models used the same outcome and predictor variables previously described with the exception of the number of mass treatments, which was excluded as a predictor variable in the model. Separate models were run for both outcomes and at each level of mass treatment.” |
| Results | | |
| Participants | 13* | Pg. 10-12  (a) “Data from the Togo Ministry of Health surveys were available for 33,363 students, including 16,473 students from 1,129 schools in 2009 and 16,890 students from 1,126 schools in 2015 (Table 1). The number of schools surveyed at baseline (2009) and at follow-up remained nearly constant for each of the five regions. Data on hookworm infection was available for nearly all children surveyed, including 16,090 (97.7%) students at baseline and 16,887 (>99.9%) at follow-up.”  (See tables for applicable n values) |
|  |  | (b) not applicable |
|  |  | (c) not applicable |
| Descriptive data | 14* | (a) Pg. 10-13, Tables 1-3 |
|  |  | (b) See Tables 2-3 |
| Outcome data | 15* | See Table 2 |
| Main results | 16 | (*a*) Pg. 13-15, Table 4  “Several school WASH conditions were associated with odds of infection (presence/absence of eggs) and particularly with intensity of infection (EPG of stool, Table 4). Compared to no drinking water at school, availability of unimproved drinking water was associated with higher odds of infection and intensity of infection (OR = 1.40, 95% CI: 1.09-1.80; EPG ratio = 1.20, 95% CI: 1.13-1.28). Access to improved drinking water, either off or on school grounds, was not associated with the prevalence of hookworm compared to no drinking water (OR = 0.90, 95% CI: 0.71-1.14; OR 1.14, 95% CI: 0.92-1.42). We found that access to improved drinking water off school grounds was associated with lower intensity of infection compared to no access (IRR = 0.80, 95% CI: 0.76-0.85), whereas having access to improved drinking water on school grounds was associated with higher intensity of infection (EPG ratio = 1.50, 95% CI: 1.42-1.58). Neither handwashing stations (with water OR = 0.85, 95% CI: 0.57-1.29; with water and soap/ash OR = 0.77, 95% CI: 0.57-1.05) nor latrine access at school (sex separate, non-private latrine OR = 0.84, 95% CI: 0.52-1.36; sex separate, private latrine OR = 0.93, 95% CI: 0.77-1.14) was associated with prevalence of infection. In contrast, having a handwashing station with water (and no soap/ash) and having access to a sex separate, private latrine was associated with lower intensity of infection (EPG ratio = 0.79, 95% CI: 0.71-0.87; EPG ratio = 0.90, 95% CI: 0.86-0.95)… Among the community-level WASH variables explored, neither improved drinking water nor latrine coverage was included in the models due to multicollinearity.” |
|  |  | (*b*) See Table 2 |
|  |  | (*c*) not applicable |
| Other analyses | 17 | Pg 15-16  “The significance of the 2009 prevalence of hookworm motivated several sensitivity analyses performed on subsets of the population. When limited to students at schools with a low prevalence of hookworm infection at baseline… Among students at schools with a high prevalence of infection at baseline…”  “Multivariable associations were assessed among children at schools that neither received community-based deworming treatment nor reported any school-based deworming treatment in the previous 12 months (n = 2,009).” |
| Discussion | | |
| Key results | 18 | Pg. 16  “Results from the 2015 assessment indicate WASH conditions were poor for a majority of schools. Few WASH conditions were found to be significant predictors of the presence or absence of hookworms in an individual, however, intensity of infection was associated with several individual, school and community-level WASH characteristics, although the direction of the association was inconsistent.” |
| Limitations | 19 | Pg. 19-20  “Limitations of the included variables prevented more detailed analysis of the effect of treatment. The variables included in the analysis do not take into account the timing of treatments and the outcome variables do not capture change in hookworm prevalence or intensity of infection. Such outcome assessment was not possible at the individual pupil level because the students from the original baseline evaluation were not the same as those surveyed at follow-up, prohibiting linkage of pre- and post-treatment data.”  “Other considerable constraints of the study should be noted. The observational nature of the study and inability to measure hookworm in the same individuals at the surveyed schools over time restricts causal inference. Non-random selection of students and the narrow age range of the students is of concern, potentially limiting how well the underlying endemicity of hookworm at each school was captured, the representativeness of follow-up study participants and the study’s generalizability within Togo. Data collection constraints also prevented evaluation of several important confounders, such as the number of students per school, un-programmed deworming activities, WASH practices or WASH conditions at baseline and other measures of contact with soil. Estimates of community-level characteristics (water source and latrine use) are limited in that they were extrapolated from a small number of students’ reported household characteristics and should be interpreted cautiously. Lastly, this analysis does not take into effect the potential impact of mass treatments for lymphatic filariasis received in several communities prior to the 2009 survey possibly diluting our measures of the association between hookworm and WASH conditions.” |
| Interpretation | 20 | Pg. 20-21  “This school-based cross-sectional study examination of the association between hookworm and WASH conditions in the context of a community receiving mass chemotherapy treatment provides insight into the impact of WASH on hookworm in the context of mass preventive chemotherapy. These findings emphasize the complex, often unpredictable relationship between WASH and hookworm. The role of school WASH conditions on hookworm infection and burden varied and often depended on the underlying endemicity of hookworm infection, with more WASH characteristics being associated with infection and disease burden among children in schools with a high underlying endemicity.” |
| Generalisability | 21 | Pg. 19  “Non-random selection of students and the narrow age range of the students is of concern, potentially limiting how well the underlying endemicity of hookworm at each school was captured, the representativeness of follow-up study participants and the study’s generalizability within Togo.” |
| Other information | | |
| Funding | 22 | Pg. 24  “This work was funded by the United States Agency for International Development through the End in Africa program at FHI360 and Health and Development International. Time contributed to the project by Julia Baker was funded by a PhD studentship stipend from the Laney Graduate School, Emory University. The funders had no role in study design, data collection and analysis, decision to publish, or preparation of the manuscript.” |
