# Supplementary material for: The associations between water and sanitation and hookworm infection using cross-sectional data from Togo's national deworming program
Source: PLoS Negl Trop Dis. 2018 Mar 28;12(3):e0006374. doi: 10.1371/journal.pntd.0006374 (PMC5902041; doi:10.1371/journal.pntd.0006374)
Supplement: S1 Model Statements — (DOCX) [file pntd.0006374.s002.docx]

**S1 Model Statements.** General model statements for prevalence and intensity of hookworm infection.

Logistic model for prevalence of hookworm infection:

Logit P(Y_ij_) = α + β_1_X_ij1_ + … + β_n_X_ijn_

Where:

Y ~ Binomial

P(Y_ij_) = Probability of hookworm infection for the j^th^ student in the i^th^ school

α = intercept

β_n_ = fixed effects for each of the n predictors

X_ij1…n_ = WASH conditions 1 through n for the j^th^ student in the i^th^ school

Negative binomial model for intensity of hookworm infection:

Log(Y_ij_) = α + β_1_X_ij1_ + … + β_n_X_ijn_

Where:

Y ~ Negative binomial

Y_ij_ = Hookworm EPG for the j^th^ student in the i^th^ school

α = intercept

β_n_ = fixed effects for each of the n predictors

X_ij1…n_ = WASH conditions 1 through n for the j^th^ student in the i^th^ school
