# Supplementary material for: The associations between water and sanitation and hookworm infection using cross-sectional data from Togo's national deworming program
Source: PLoS Negl Trop Dis. 2018 Mar 28;12(3):e0006374. doi: 10.1371/journal.pntd.0006374 (PMC5902041; doi:10.1371/journal.pntd.0006374)
Supplement: S1 Table — (DOCX) [file pntd.0006374.s003.docx]

**S1 Table.** Multivariable associations between WASH conditions and prevalence of hookworm infection (presence/absence of eggs in stool), stratified by number of house-to-house deworming treatments

|  | **Children that received 0-1**  **house-to-house deworming treatments** | | | **Children that received 4-5**  **house-to-house deworming treatments** | | | **Children that received 7-8**  **house-to-house deworming treatments** | | |
| --- | --- | --- | --- | --- | --- | --- | --- | --- | --- |
| **Condition of interest** | **OR** | **95% CI** | **p-value** | **OR** | **95% CI** | **p-value** | **OR** | **95% CI** | **p-value** |
| **WASH conditions** | | | | | | |  |  |  |
| Water source and availability at school (categorical) | | | | | | |  |  |  |
| No drinking water available* | ref | -- | -- | -- | -- | -- | -- | -- | -- |
| Unimproved drinking water available | 1.00 | 0.49, 2.05 | 1.00 | 1.53 | 1.15, 2.02 | **<0.01** | 0.98 | 0.41, 2.37 | 0.97 |
| Improved drinking water available, not on  school grounds | 1.15 | 0.63, 2.11 | 0.65 | 0.90 | 0.70, 1.18 | 0.43 | 1.32 | 0.72, 2.43 | 0.37 |
| Improved drinking water available, on  school grounds | 1.85 | 1.05, 3.27 | **0.03** | 1.20 | 0.93, 1.55 | 0.16 | 1.11 | 0.66, 1.85 | 0.70 |
| Handwashing station availability at school (categorical) | | | | | | |  |  |  |
| No handwashing station or station without  water* | ref | -- | -- | -- | -- | -- | -- | -- | -- |
| Handwashing station available with water | 0.75 | 0.28, 2.01 | 0.56 | 0.77 | 0.47, 1.26 | 0.29 | 1.75 | 0.59, 5.22 | 0.32 |
| Handwashing station available with water  and soap/ash | 0.73 | 0.26, 2.02 | 0.54 | 0.75 | 0.54, 1.04 | 0.09 | 0.16 | 0.02, 1.67 | 0.13 |
| Latrine availability and type at school (categorical) | | | | | | |  |  |  |
| No latrine or not sex separate* | ref | -- | -- | -- | -- | -- | -- | -- | -- |
| Sex separate, non-private latrine available | 1.93 | 0.24, 15.63 | 0.54 | 0.74 | 0.44, 1.23 | 0.24 | Omitted because of collinearity | | |
| Sex separate, private latrine available | 1.08 | 0.64, 1.83 | 0.77 | 0.90 | 0.71, 1.14 | 0.40 | 1.04 | 0.67, 1.61 | 0.87 |
| Child wearing shoes | 1.00 | 0.69, 1.43 | 0.98 | 0.64 | 0.56, 0.73 | **<0.01** | 0.71 | 0.50, 1.00 | 0.05 |
| **School-level background variables** | | | | | | |  |  |  |
| 2009 hookworm prevalence | 4.66 | 1.43, 15.22 | **0.01** | 5.90 | 4.02, 8.66 | **<0.01** | 4.62 | 1.89, 11.30 | **<0.01** |
| Deworming treatment in the last 12 months | 0.91 | 0.56, 1.47 | 0.70 | 1.25 | 1.02, 1.52 | **0.03** | 0.96 | 0.65, 1.42 | 0.85 |

*Reference category. Models control for 2015 population density, distance from school to water, district, and land cover.

Bold p-values are statistically significant at the α = 0.05 level
