# Supplementary material for: The associations between water and sanitation and hookworm infection using cross-sectional data from Togo's national deworming program
Source: PLoS Negl Trop Dis. 2018 Mar 28;12(3):e0006374. doi: 10.1371/journal.pntd.0006374 (PMC5902041; doi:10.1371/journal.pntd.0006374)
Supplement: S2 Table — (DOCX) [file pntd.0006374.s004.docx]

**S2 Table.** Multivariable associations between WASH conditions and intensity of hookworm infection (eggs per gram of stool), stratified by number of house-to-house deworming treatments

|  | **Children that received 4-5**  **house-to-house deworming treatments** | | | **Children that received 7-8**  **house-to-house deworming treatments** | | |
| --- | --- | --- | --- | --- | --- | --- |
| **Condition of interest** | **EPG ratio** | **95% CI** | **p-value** | **EPG ratio** | **95% CI** | **p-value** |
| **WASH conditions** | | | | | | |
| Water source and availability at school (categorical) | | | | | | |
| No drinking water available* | ref | -- | -- | -- | -- | -- |
| Unimproved drinking water available | 1.10 | 1.02, 1.19 | **0.01** | 0.73 | 0.59, 0.91 | **<0.01** |
| Improved drinking water available, not on  school grounds | 0.70 | 0.66, 0.75 | **<0.01** | 0.69 | 0.59, 0.81 | **<0.01** |
| Improved drinking water available, on  school grounds | 1.46 | 1.37, 1.55 | **<0.01** | 1.47 | 1.30, 1.66 | **<0.01** |
| Handwashing station availability at school (categorical) | | | | | | |
| No handwashing station or station without  water* | ref | -- | -- | -- | -- | -- |
| Handwashing station available with water | 0.75 | 0.67, 0.84 | **<0.01** | 3.63 | 2.66, 4.97 | **<0.01** |
| Handwashing station available with water  and soap/ash | 0.96 | 0.90, 1.03 | 0.29 | 0.03 | 0.02, 0.05 | **<0.01** |
| Latrine availability and type at school (categorical) | | | | | | |
| No latrine or not sex separate* | ref | -- | -- | -- | -- | -- |
| Sex separate, non-private latrine available | 0.91 | 0.81, 1.04 | 0.17 | Omitted because of collinearity | | |
| Sex separate, private latrine available | 0.87 | 0.82, 0.92 | **<0.01** | 0.92 | 0.82, 1.02 | 0.13 |
| Child wearing shoes | 0.44 | 0.42, 0.46 | **<0.01** | 0.28 | 0.25, 0.31 | **<0.01** |
| **School-level background variables** | | | | | | |
| 2009 hookworm prevalence | 7.24 | 6.56, 8.00 | **<0.01** | 2.97 | 2.38, 3.69 | **<0.01** |
| Deworming treatment in the last 12 months | 1.24 | 1.18, 1.30 | **<0.01** | 0.59 | 0.54, 0.66 | **<0.01** |

*Reference category. Models control for 2015 population density, distance from school to water, district, and land cover.

Bold p-values are statistically significant at the α = 0.05 level

Results are not presented for children that received 0-1 deworming treatments due to non-convergence of the model.
